# Neural circuitry at age 6 months associated with later repetitive behavior and sensory responsiveness in autism

**DOI:** 10.1186/s13229-017-0126-z

**Published:** 2017-03-04

**Authors:** Jason J. Wolff, Meghan R. Swanson, Jed T. Elison, Guido Gerig, John R. Pruett, Martin A. Styner, Clement Vachet, Kelly N. Botteron, Stephen R. Dager, Annette M. Estes, Heather C. Hazlett, Robert T. Schultz, Mark D. Shen, Lonnie Zwaigenbaum, Joseph Piven, The IBIS Network, J. Piven, H. C. Hazlett, S. Dager, A. Estes, D. Shaw, K. Botteron, R. McKinstry, J. Constantino, J. Pruett, R. Schultz, S. Paterson, L. Zwaigenbaum, J. Elison, A. C. Evans, D. L. Collins, G. B. Pike, V. Fonov, P. Kostopoulos, S. Das, G. Gerig, M. Styner, H. Gu

**Affiliations:** 1 Department of Educational Psychology, University of Minnesota, Minneapolis, MN USA; 2 Carolina Institute for Developmental Disabilities, University of North Carolina, Chapel Hill, NC USA; 3 Institute of Child Development, University of Minnesota, Minneapolis, MN USA; 4 Tandon School of Engineering, New York University, New York City, NY USA; 5 Department of Psychiatry, Washington University School of Medicine, St. Louis, MO USA; 6 Department of Psychiatry, University of North Carolina, Chapel Hill, NC USA; 7 Scientific Computing and Imaging Institute, University of Utah, Salt Lake City, UT USA; 8 Department of Radiology, University of Washington, Seattle, WA USA; 9 Department of Speech and Hearing Sciences, University of Washington, Seattle, WA USA; 10 Center for Autism Research, Children’s Hospital of Philadelphia, Philadelphia, PA USA; 11 Department of Pediatrics, University of Alberta, Edmonton, AB Canada

**Keywords:** Infant, Diffusion tensor imaging, Autism, Repetitive behavior, White matter, Longitudinal

## Abstract

**Background:**

Restricted and repetitive behaviors are defining features of autism spectrum disorder (ASD). Under revised diagnostic criteria for ASD, this behavioral domain now includes atypical responses to sensory stimuli. To date, little is known about the neural circuitry underlying these features of ASD early in life.

**Methods:**

Longitudinal diffusion tensor imaging data were collected from 217 infants at high familial risk for ASD. Forty-four of these infants were diagnosed with ASD at age 2. Targeted cortical, cerebellar, and striatal white matter pathways were defined and measured at ages 6, 12, and 24 months. Dependent variables included the Repetitive Behavior Scale-Revised and the Sensory Experiences Questionnaire.

**Results:**

Among children diagnosed with ASD, repetitive behaviors and sensory response patterns were strongly correlated, even when accounting for developmental level or social impairment. Longitudinal analyses indicated that the genu and cerebellar pathways were significantly associated with both repetitive behaviors and sensory responsiveness but not social deficits. At age 6 months, fractional anisotropy in the genu significantly predicted repetitive behaviors and sensory responsiveness at age 2. Cerebellar pathways significantly predicted later sensory responsiveness. Exploratory analyses suggested a possible disordinal interaction based on diagnostic status for the association between fractional anisotropy and repetitive behavior.

**Conclusions:**

Our findings suggest that restricted and repetitive behaviors contributing to a diagnosis of ASD at age 2 years are associated with structural properties of callosal and cerebellar white matter pathways measured during infancy and toddlerhood. We further identified that repetitive behaviors and unusual sensory response patterns co-occur and share common brain-behavior relationships. These results were strikingly specific given the absence of association between targeted pathways and social deficits.

**Electronic supplementary material:**

The online version of this article (doi:10.1186/s13229-017-0126-z) contains supplementary material, which is available to authorized users.

## Background

Restricted and repetitive behaviors (RRBs) are defining characteristics of autism spectrum disorder (ASD). Behaviors comprising this domain range from relatively simple topographies—such as motor stereotypies—to more complex forms including inflexible adherence to routines and intense, circumscribed interests. There is evidence that RRBs in toddlerhood are early emerging, prognostic features [1] that may differentiate infants who do and do not later develop ASD [2, 3]. Separately, neuroimaging studies of infant siblings of children with ASD, who are themselves at elevated risk for the disorder, indicate that atypical features and trajectories of brain development may be evident as early as 6 months of age in children who later receive a diagnosis [4–7]. Although such brain changes occur in parallel with emerging patterns of atypical RRBs, the specific aspects of brain development underlying their emergence in infancy is unknown.

Preclinical work has implicated components of cortico-striatal-thalamo-cortical circuitry [8] as the neurobiological basis of RRBs [9–11]. Specific to individuals with ASD, there is supporting evidence linking RRBs with connectivity and morphology of this system in children [12–15] and adults [16–18]. Despite some consistency across studies, the direction of effects within the same structures or circuits is mixed. For example, striatal volumes have been reported to be positively [13, 14] and negatively [12, 19] correlated with repetitive behavior. What appear as inconsistent findings may instead reflect phenotypic heterogeneity or developmental effects, wherein the role of striatal circuits and structures in the etiology and maintenance of RRBs is not static across subpopulations or over time [13, 14, 20].

Sensorimotor processing and motor control are also supported by the cerebellum [21–23]. Studies implicating the cerebellum in ASD extend back over two decades [24–26], and this structure has been linked to RRBs associated with the disorder in humans and nonhuman animal models [18, 27–29]. Repetitive behavior has been inversely correlated with cerebellar volume among adults with ASD [18], and cerebellar hypoplasia has been linked to stereotypy and decreased environmental exploration in children [28]. Similar results have been reported more recently with the addition of significant positive associations between RRBs and vermis grey matter [27].

Recently updated nosology of ASD under DSM 5 includes for the first time symptoms related to unusual behavioral responses to, or interests in, sensory stimuli [30] as part of restricted and repetitive behaviors. While this change reflects clinical consensus, the conceptual grouping of RRBs with unusual responses to sensory stimuli in ASD is supported by a limited body of empirical and theoretical work addressing the relationship between these features of ASD [30–33]. There is evidence that as with RRBs, atypical sensory responses are evident in toddlerhood [34, 35] and are linked to patterns of neural connectivity in adolescence [36, 37]. While not yet empirically tested, it has been hypothesized that early cerebellar dysfunction may also explain the range of sensory response patterns observed in ASD [38, 39].

Our primary aim was to examine the structural properties of select neural circuits in relation to RRBs and sensory responsiveness in familial high-risk infants who developed ASD. Our analyses focused on pathways connecting brain regions implicated by previous studies of RRBs, including: (1) thalamo-cortical and cortico-striatal circuitry, (2) ponto-cerebellar and cerebello-thalamic circuitry, and (3) anterior corpus callosum [5, 12–19, 27–29]. We posited that RRBs and sensory response patterns would covary in toddlers with ASD. Proceeding from this, we investigated: (1) whether development of targeted white matter pathways, measured from infancy through toddlerhood using diffusion tensor magnetic resonance imaging (DT-MRI), would be associated with RRBs and sensory responsiveness at age 2 years; (2) whether variation in white matter pathways at age 6 months would be associated with later RRBs or sensory responsiveness at age 2 years; and (3) whether classes of behavior (i.e., repetitive behaviors and sensory responsiveness) were characterized by overlapping versus distinct brain-behavior relationships. As a follow-up to these aims, we examined whether observed effects extended to high-risk infants who did not receive a diagnosis of ASD.

## Methods

### Participants

Participants were from the Infant Brain Imaging Study, a prospective, longitudinal study of infants at high and low familial risk for ASD. Familial high-risk status was defined by having an older sibling with a community diagnosis of the ASD confirmed by the Autism Diagnostic Interview-Revised (ADI-R) [40] and Social Communication Questionnaire [41]. Infants were enrolled at one of four clinical data collection sites: the Children’s Hospital of Philadelphia, University of North Carolina, University of Washington, and Washington University in St. Louis. Exclusion criteria were (1) evidence of a genetic condition or condition affecting development; (2) significant vision or hearing impairment; (3) birth weight < 2000 g or gestational age < 36 weeks; (4) significant perinatal adversity or prenatal exposure to neurotoxins, (5) contraindication for MRI, (6) predominant home language other than English, (7) adopted, half siblings, or twins and (8) first degree relative with psychosis, schizophrenia, or bipolar disorder.

The present sample included high-risk children who met the following criteria: (1) at least one complete DT-MRI scan and (2) complete cognitive and behavioral assessment battery at age 2 years including diagnostic evaluation and assessment of RRBs. Our primary sample of interest were high-risk infants who received a clinical best-estimate diagnosis of ASD at age 2 years (HR-ASD; *n* = 44). We also included high-risk infants who did not receive a diagnosis of ASD at age 2 (HR-Neg; *n* = 173) to discern whether effects observed among HR-ASD extended to unaffected children with shared familial liability for ASD. Clinical best-estimate diagnoses were made based upon DSM-IV-TR criteria using all available assessment data including the Autism Diagnostic Observation Schedule (ADOS) [42], ADI-R, Mullen Scales of Early Learning (MSEL) [43], and the Vineland Adaptive Behavior Scales II [44]. Reliability for these standardized instruments was initially established and maintained between sites through monthly case reviews. Clinical best-estimate diagnoses made at age 2 years were independently confirmed by a second senior clinician blind to risk status and diagnosis made by the first clinician. All participants in our study sample had a completed ADOS administration. MSEL data were incomplete for two participants (1 HR-ASD, 1 HR-Neg), and composite scores from age 12 months were substituted. Although the parent study also collects data on low-familial risk infants, this group was excluded from the present study given lack of variance in RRB scores due to floor effect [3]. Written informed consent was obtained for all participants from their parent or guardian, and all study procedures were approved by institutional review boards at each clinical site (Children’s Hospital of Philadelphia, University of North Carolina, University of Washington at St. Louis, and Washington University).

### Clinical measures

Assessment data included the Repetitive Behavior Scale-Revised (RBS-R) [45] and the Sensory Experiences Questionnaire v2.1 (SEQ) [46] administered at age 2 years. The RBS-R is a measure of RRBs consisting of 43 items, each of which represents a discrete behavioral topography. The RBS-R is sensitive to individual differences among toddlers at high-risk for ASD [3], and the measure has been independently validated for use in children as young as age 2 years [42]. We partitioned total inventory scores from the RBS-R into two categories: *lower-order* (combining stereotypical, self-injurious, and restricted behaviors), and *higher-order* (combining compulsive, ritualistic, and sameness behaviors). This grouping was made upon the basis of conceptual and factor analytic work [47, 48].

The SEQ consists of 38 items assessing responses to sensory stimuli across modalities. In addition to a total score, the SEQ yields summary scores for items representing hypo- and hyper-responsivity and sensory seeking. The SEQ has been shown to have strong psychometric properties [46, 49]. Complete SEQ data was available for 89% (*n* = 39) of the HR-ASD sample and 83% (*n* = 144) of the HR-Neg sample. The MSEL Early Learning Composite (MSEL composite) [43] was used to characterize general developmental level. Social affect, RRB, and total severity scores were derived from the Autism Diagnostic Observation Schedule (ADOS) [42]. Descriptive data for the study sample are provided in Table 1.

**Table 1:** Descriptive data for study sample

Variable	HR-ASD	HR-Neg	*P* ^a^
Six-month sample	32	106	
Total longitudinal sample	44	173	
Longitudinal scan complement			
6, 12, and 24 m scan	15	54	
6 and 12 m scan	7	28	
6 and 24 m scan	6	11	
12 and 24 m scan	7	37	
6 m scan	4	13	
12 m scan	3	22	
24 m scan	2	8	
Mean age time 1	6.4 (0.4)	6.7 (0.7)	.08
Mean age time 2	12.8 (0.7)	12.6 (0.6)	.16
Mean age time 3	24.5 (0.7)	24.7 (0.7)	.92
Sex (% male)	89	58	<.001
ADOS repetitive behavior	2.7 (1.8)	0.7 (0.9)	<.001
ADOS social affect	12.0 (3.8)	2.3 (2.2)	<.001
ADOS severity score	6.0 (1.9)	1.6 (1.0)	<.001
MSEL composite	79.3 (16.9)	102.1 (15.8)	<.001
RBS-R total items endorsed	6 (0–38)^b^	2 (0–27)^b^	<.001
SEQ total raw score	51.7 (12.6)	39.8 (7.6)	<.001

^a^Test statistic for independent samples *t* test except for sex (Fisher’s exact test) and RBS-R total items endorsed (Mann-Whitney *U* test)

^b^Median and range

*ADOS* Autism Diagnostic Observation Schedule, *MSEL* Mullen Scales of Early Learning, *RBS-R* Repetitive Behavior Scale-Revised, *SEQ* Sensory Experiences Questionnaire

### MRI data acquisition and processing

MRI scans were acquired on 3T Siemens TIM Trio scanners equipped with 12-channel head coils during natural sleep. The imaging protocol included: sagittal T1 MP-Rage (TR = 2400 ms, TE = 3.16 ms, slice thickness = 1 mm, FOV = 256 mm, 256 × 160 matrix), 3D T2 fast spin echo (TR = 3200 ms, TE = 499 ms, slice thickness = 1 mm, FOV = 256 mm, 256 × 160 matrix), and 25-direction ep2d_diff with FoV = 190 mm (6 and 12 months) or FoV = 209 mm (24 months), 75–81 transversal slices, slice thickness = 2 mm isotropic, 2 × 2 × 2 mm3 voxel resolution, TR = 12,800–13,300 ms, TE = 102 ms, variable *B* value 0–1000 s/mm^2^. Intra- and inter-site reliability was established and maintained across sites and time [50].

Diffusion-weighted images were processed with DTIprep, which detects common artifacts, corrects for motion and eddy current deformations, and flags bad gradients for manual removal by expert raters [51, 52]. Data sets with fewer than 18 (of 25 total) gradients were excluded from further processing to ensure consistent signal-to-noise ratio. Overall, approximately 10.5% of acquired DTI datasets were excluded following quality control procedures, with about one quarter of excluded cases due to subject motion. An additional 4% of all DWI scans were excluded for incomplete acquisition (e.g., child awoke during scan). There were no differences between HR children with and without a diagnosis of ASD in proportion of scans excluded. Group analysis of diffusion weighted data utilized an atlas-based processing pipeline providing consistent spatial parameterization within and between individual datasets across ages [53, 54].

**Fig. 1:**
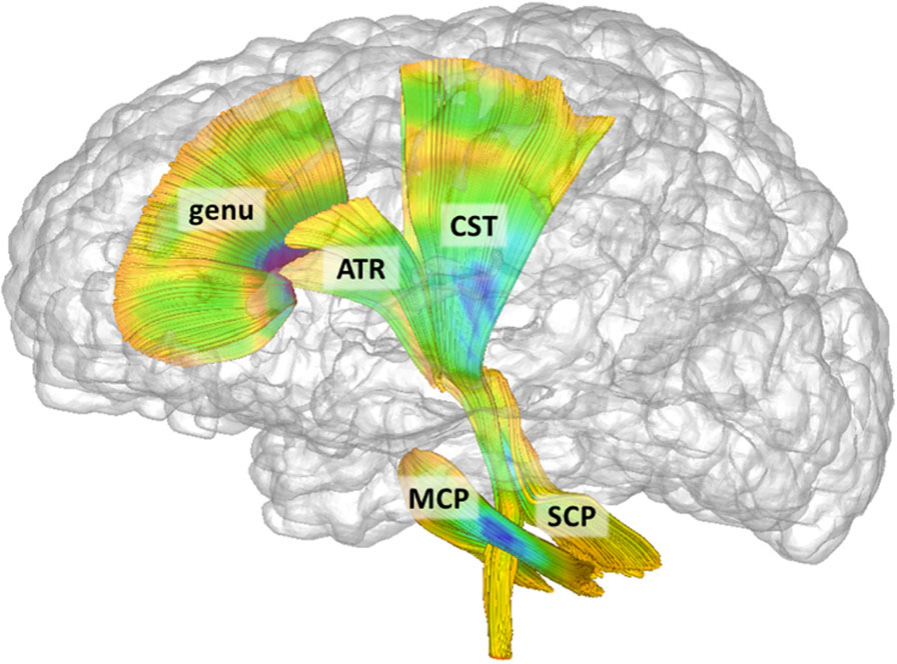
White matter fiber pathways defined through tractography of DTI data. Targeted pathways include *ATR* anterior thalamic radiation, *CST* cortico-spinal tract, *genu* genu of corpus callosum, *MCP* mid-cerebellar peduncle, *SCP* superior cerebellar peduncle

Deterministic fiber tractography was performed by the first and second author in the study specific, average atlas space using 3D Slicer (https://www.slicer.org/) and refined via FiberViewerLight. Fiber tract definitions followed anatomically informed methods [55]. Pathways of interest (Fig. 1) included the cortical-spinal tract (CST), which passes through the basal ganglia to the motor-sensory cortex; anterior thalamic radiation (ATR), which passes from thalamus to pre-frontal cortex; genu of the corpus callosum, which connects frontal hemispheres; mid-cerebellar peduncle (MCP), which passes through the pons and connects cerebellar hemispheres; and the superior cerebellar peduncles (SCP), which connect the cerebellum and thalamus.

Fractional anisotropy (FA) values were obtained via DTIAtlasFiberAnalyzer[53]. FA represents degree of diffusion along the primary fiber orientation (axial diffusivity) relative to transverse diffusion (radial diffusivity). Following previous infant DTI studies [56, 57], bilateral pathways were averaged to yield a single estimate. All components of this processing pipeline are freely available as part of the UNC/Utah NAMIC DTI Fiber Analysis Framework [53].

### Statistical analysis

Because RBS-R and SEQ scores were non-normally distributed, Spearman’s rank order correlations were used to examine strength of association between RRBs and sensory responsiveness. Correlation analyses were performed using bootstrap sampling with replacement (*B* = 1000) to generate confidence interval estimates. Follow-up analyses were conducted with control for possible confounding variables: ADOS social affect and MSEL composite. Correlation analyses were uncorrected for multiple comparisons with α = .05.

We next examined longitudinal imaging data across ages 6, 12, and 24 months in relation to these behaviors using generalized estimating equations (GEE). Predictors included fractional anisotropy of target white matter pathways, sex, and age. MSEL composite score was assessed as a potential covariate and ultimately excluded from our analytic models as this variable did not contribute to model fit and was not itself significantly associated RBS-R or SEQ scores. Sex and age were included as covariates in all models given potential effects on DTI data [54]. The interaction of FA and age was tested for each model to determine whether differences in slope explained the relationship between FA and dependent variables. Secondary analysis was performed using subscales derived from the RBS-R and SEQ and all targeted fiber pathways to fully explore possible brain-behavior relationships.

Models were fit based upon response distribution and measurement type of the primary dependent variables. For analyses of RRBs, a negative binomial regression model with log link function was fit with total RBS-R inventory as the dependent variable. This approach was selected given that the RBS-R inventories constituted integer count data. Goodness-of-fit statistics indicated that a negative binomial distribution was superior to a Poisson distribution. For sensory responsiveness, a gamma regression model with log link function was fit with SEQ total raw score as the dependent variable. This approach was selected given that SEQ data followed a log-normal distribution. Goodness-of-fit statistics indicated that this was superior to regression based on a Gaussian distribution. Secondary analyses were performed on subscale measures derived from the RBS-R and SEQ and included higher- and lower-order repetitive behaviors (from RBS-R) and hypo- and hyper-responsiveness and sensory seeking (from SEQ) as dependent variables following significant primary model results. Exponentiated beta (*e* ^B^) coefficients were generated to provide an estimate of effect size (incident rate ratio).

Following longitudinal analyses, we next employed generalized linear modeling (GLM) to focus specifically on the predictive relationship of white matter pathways measured at age 6 months with RRBs and sensory responsiveness measured at age 2 years. These models were controlled for the effects of sex and age and were fit based on the response distributions and link functions described above. In a final set of exploratory analyses, we replicated the steps described above in high-risk infants who did not meet diagnostic criteria for ASD at age 2 years (HR-Neg) to determine if effects observed in HR-ASD extended to unaffected siblings. An adaptive false-discovery rate procedure was applied at each stage of primary and secondary GEE and GLM analyses to account for multiple comparisons [58]. Adjusted *q* values are presented with the significance threshold at *q* < 0.05. Supplementary analyses were conducted to explore the differential roles of axial and radial diffusivities, as well as possible lateralization effects (see Additional files). Results from supplementary and exploratory analyses are presented without correction for multiple comparisons.

## Results

### Concurrence of repetitive behavior and sensory response patterns

At 24 months, RBS-R scores correlated strongly with total SEQ, *r*_s_ = 0.74, *p* < 0.001, 95% CI [0.55, 0.87], supporting our hypothesis that RRBs and sensory responsiveness would covary in the HR-ASD group. This correlation held up to partial models controlling for MSEL composite (pr_s_ = 0.75, *p* < 0.001) and ADOS social affect (pr_s_ = 0.74, *p* < 0.001). Correlations of lower- and higher-order RRBs derived from the RBS-R with patterns of sensory responsiveness from the SEQ (hypo, hyper, and seeking) were all statistically significant with the exception of the association between higher-order RRB and sensory seeking behavior (see Table 2).

**Table 2:** Nonparametric correlations for subscales derived from the RBS-R and SEQ among HR-ASD

	SEQ total	SEQ hypo	SEQ hyper	SEQ Sensory Seeking
RBS-R lower-order				
*r*_s_	0.75***	0.67***	0.57***	0.53**
95% CI	0.57, 0.86	0.45, 0.80	0.30, 0.74	0.24, 0.74
RBS-R higher-order				
*r*_s_	0.53**	0.50**	0.39*	0.21
95% CI	0.12, 0.74	0.18, 0.71	0.04, 0.65	−0.14, 0.54

*RBS-R* Repetitive Behavior Scale-Revised, *SEQ* Sensory Experiences Questionnaire

***p < 0.001, **p < 0.01, *p < .05

### Longitudinal brain development in relation to behavior at age 2

To expand on the results from age 6 months, we next examined development of FA in target pathways across 6, 12, and 24 months in HR-ASD in relation to total RBS-R and SEQ. These results are presented in Table 3. For the RBS-R, there were significant main effects for the genu, MCP, and SCP over the 6 to 24-month interval. These effects were largely consistent across lower- and higher-order RRBs derived from the RBS-R (Table 4). There were no significant effects for the ATR or CST.

**Table 3:** Longitudinal model results for effect of fractional anisotropy on total RBS-R and SEQ among HR-ASD

Variable	χ^2^	*q*	*e* ^B^	CI
Total RBS-R^1^				
ATR FA	3.2	0.06	1.07	0.99–1.16
CST FA	0.2	0.31	1.02	0.94–1.10
Genu FA	16.3	<0.001	1.14	1.07–1.22
MCP FA	8.3	0.004	1.07	1.02–1.12
SCP FA	7.0	0.006	1.12	1.03–1.22
Total SEQ^1^				
ATR FA	0.9	0.18	1.01	0.99–1.04
CST FA	0.0	0.38	1.00	0.98–1.02
Genu FA	13.0	<0.001	1.04	1.02–1.06
MCP FA	9.5	0.003	1.02	1.01–1.04
SCP FA	6.8	0.006	1.03	1.01–1.06

Generalized estimating equation model results

*RBS-R* Repetitive Behavior Scale-Revised, *SEQ* Sensory Experiences Questionnaire, *ATR* anterior thalamic radiation, *CST* cortico-spinal tract, *SCP* superior cerebellar peduncle, *MCP* midcerebellar peduncle

*q* false-discovery rate adjusted *p* value, *e* ^*B*^ exponentiated regression coefficient representing percent of increase in score for every hundredth unit increase in fractional anisotropy, *CI* 95% confidence interval

**Table 4:** Secondary longitudinal analysis results for fractional anisotropy and behavior for HR-ASD

Variable	χ^2^	*q*	*e* ^B^	CI
RBS-R				
Lower order				
ATR FA	1.2	0.33	1.05	0.96–1.14
CST FA	0.0	0.84	1.01	0.93–1.09
Genu FA	5.7	0.05	1.09	1.02–1.17
MCP FA	6.2	0.048	1.07	1.01–1.12
SCP FA	6.7	0.04	1.13	1.03–1.23
Higher order				
ATR FA	4.2	0.08	1.10	1.00–1.20
CST FA	0.6	0.42	1.04	0.95–1.13
Genu FA	25.3	<0.001	1.21	1.12–1.30
MCP FA	6.9	0.04	1.08	1.02–1.14
SCP FA	4.4	0.07	1.11	1.01–1.23
SEQ				
Hypo				
ATR FA	0.7	0.42	1.01	0.98–1.05
CST FA	0.0	0.86	1.00	0.97–1.03
Genu FA	14.3	0.002	1.05	1.02–1.07
MCP FA	11.5	0.007	1.03	1.01–1.05
SCP FA	5.0	0.07	1.04	1.01–1.07
Hyper				
ATR FA	0.7	0.42	1.01	0.99–1.04
CST FA	0.1	0.75	1.00	0.98–1.03
Genu FA	4.5	0.07	1.02	1.00–1.05
MCP FA	2.1	0.24	1.01	1.00–1.03
SCP FA	4.9	0.07	1.03	1.00–1.06
Seeking				
ATR FA	1.3	0.33	0.98	0.93–1.02
CST FA	1.1	0.35	0.98	0.94–1.02
Genu FA	1.6	0.31	1.02	0.99–1.06
MCP FA	1.3	0.33	1.02	0.99–1.04
SCP FA	2.6	0.19	1.04	0.99–1.09

Results for generalized estimating equations

*RBS-R* Repetitive Behavior Scale-Revised, *SEQ* Sensory Experiences Questionnaire, *ATR* anterior thalamic radiation, *CST* cortico-spinal tract, *SCP* superior cerebellar peduncle, *MCP* midcerebellar peduncle

*q* false-discovery rate adjusted *p* value, *e* ^*B*^ exponentiated regression coefficient representing percent of increase in score for every hundredth unit increase in fractional anisotropy, *CI* 95% confidence interval

For total SEQ scores, there were significant main effects for the genu, MCP, and SCP (see Table 3). For subscales derived from the SEQ, there were significant effects for hypo-responsiveness in relation to the genu and MCP (Table 4). While initial analyses suggested that these pathways were likewise significantly associated with hyper-responsiveness, these results did not survive FDR correction. There were no significant main effects for the CST or ATR, and no significant effects for any pathway with Sensory Seeking. There were no additional significant effects for the interaction of Age X FA for any pathway. See Additional file 1: Table A1 for supplementary longitudinal analyses of axial and radial diffusivities.

### Specificity of longitudinal results

To assess the specificity of our findings, we first examined a control behavioral measure of a domain which we reasoned qualitatively differed from repetitive behaviors or sensory responsiveness. Namely, we conducted longitudinal analyses using social affect scores from the ADOS as our dependent variable. This revealed no significant relationships with any pathways associated with the RBS-R or SEQ (Genu FA: χ^2^ = 1.7, *p* = 0.19; MCP FA: χ^2^ = 0.0, *p* = 0.91; SCP FA: χ^2^ = 0.3, *p* = 0.59).

We next examined FA in pathways not included in our primary hypotheses to assess regional specificity. This analysis included the anterior limb of the internal capsule (ALIC) and splenium of the corpus callosum. Both have been previously implicated in imaging studies of infants at risk for ASD [6, 59] but not specifically in relation to RRBs or sensory responsiveness. The ALIC was not significantly associated with total RBS-R or SEQ scores (all *q* > 0.18). We did identify a significant main effect for the splenium and total RBS-R score (χ^2^ = 6.7, *q* = 0.08, *e* ^B^ = 1.07) but not for SEQ total score (χ^2^ = 2.6, *q* = 0.17, *e* ^B^ = 1.02).

### FA at age 6 months in relation to behavior at age 2

Generalized linear model results for HR-ASD are presented in Table 5. Genu FA at age 6 months was significantly associated with total RBS-R at age 2, (χ^2^ = 17.5, *q* < 0.001, *e* ^B^ = 1.24). For the genu, *e* ^B^ coefficients indicated that each 0.01 unit increase in FA was associated with an estimated 23% increase in total number of RBS-R items endorsed at age 2. Genu FA was significantly associated with lower-order (χ^2^ = 5.7, *q* = 0.02, *e* ^B^ = 1.16) and higher-order (χ^2^ = 32.4, *q* < 0.001, *e* ^B^ = 1.36) behaviors from the RBS-R. Each unit increase in genu FA was associated with an estimated 16 and 34% increase in count of lower- and higher-order RRBs. The other four pathways of interest were not significantly associated with total scores on the RBS-R.

**Table 5:** Fractional anisotropy at age 6 months predicting behavior at age 2 years among HR-ASD

Variable	χ^2^	*q*	*e* ^B^	CI
Total RBS-R				
ATR	0.6	0.47	1.05	0.92–1.20
CST	0.2	0.59	1.03	0.91–1.18
Genu	17.5	<0.001	1.24	1.12–1.37
SCP	0.8	0.47	1.06	0.94–1.19
MCP	1.2	0.41	1.04	0.97–1.11
Total SEQ				
ATR	2.9	0.73	1.03	1.00–1.06
CST	1.1	0.59	1.02	0.98–1.05
Genu	14.0	<0.001	1.06	1.03–1.09
SCP	2.1	0.31	1.02	0.99–1.06
MCP	4.9	0.06	1.02	1.00–1.04

Generalized linear model results

*RBS-R* Repetitive Behavior Scale-Revised, *SEQ* Sensory Experiences Questionnaire, *ATR* anterior thalamic radiation, *CST* cortico-spinal tract, *SCP* superior cerebellar peduncle, *MCP* midcerebellar peduncle

*q* false-discovery rate adjusted *p* value, *e* ^*B*^ exponentiated regression coefficient representing percent of increase in score for every hundredth unit increase in fractional anisotropy, *CI* 95% confidence interval

FA in the genu (χ^2^ = 14.0, *q* < 0.001, *e* ^B^ = 1.06) at age 6 months was significantly associated with total SEQ score at age 2 years. Each unit increase in genu FA was associated with an estimated 6% increase in SEQ score. The MCP (χ^2^ = 4.9, *p* = 0.02, *e* ^B^ = 1.02) was also associated with later SEQ score, though this result did not survive FDR correction (*q* = 0.056). Genu FA was significantly associated with sensory hypo-responsivity on the SEQ (χ^2^ = 10.0, *q* = .002, *e* ^B^ = 1.08). Hyper-responsivity scores were also significantly associated with FA in the genu (χ^2^ = 4.9, *q* = 0.014, *e* ^B^ = 1.04). Genu FA was not significantly associated with sensory seeking scores. To provide visualization of RBS-R and SEQ data in relation to FA measures, example scatterplots showing the genu and CST are provided in Additional file 2: Figure S1. See Additional file 1: Table A2 for supplementary analyses of axial and radial diffusivity.

### Unaffected high-risk siblings

For HR-Neg toddlers (*n* = 137), total RBS-R scores significantly correlated with total SEQ, *r*_s_ = 0.49, *p* < 0.001, 95% CI [0.35, 0.60]. This relationship remained when controlling for MSEL composite score (pr_s_ = 0.47, *p* < 0.001) or social affect score (pr_s_ = 0.48, *p* < 0.001). Correlations of lower- and higher-order RRB scores derived from the RBS-R with patterns of sensory responsiveness from the SEQ (hypo, hyper, and seeking) were all statistically significant (see Additional file 1: Table A3).

Results from longitudinal analyses of HR-Neg participants are presented in Table 6. There were no significant effects observed for FA in relation to total RBS-R or SEQ scores after correction for multiple comparisons. However, the uncorrected model and the effect size estimate for the genu did suggest a possible association (*p* = .035, uncorrected; *e* ^B^ = 0.94, 95% CI [0.89, 0.99]). There were no additional significant effects for the interaction of Age X FA for any pathway. Follow-up analyses conducted with the ALIC and splenium revealed no significant effects for the ALIC in relation to RBS-R or SEQ total scores (all *q* > 0.25). Splenium FA was not significantly associated with total SEQ scores (*q* = 0.99). As with the genu, uncorrected model results suggested that splenium FA was significantly associated with total RBS-R, though this result did not survive FDR correction (χ^2^ = 7.6, *q* = 0.08, *e* ^B^ = 0.95, 95% CI [0.91, 0.99]).

**Table 6:** Longitudinal model results for HR siblings not meeting diagnostic criteria for ASD

Variable	χ^2^	*q*	*e* ^B^	CI
Total RBS-R				
ATR FA	0.2	0.91	0.99	0.92–1.05
CST FA	0.8	0.66	0.97	0.91–1.04
Genu FA	4.5	0.25	0.94	0.89–0.99
MCP FA	1.5	0.46	0.98	0.94–1.01
SCP FA	2.1	0.46	0.95	0.88–1.02
Total SEQ^4^				
ATR FA	1.8	0.46	1.01	1.00–1.02
CST FA	0.0	0.99	1.00	0.99–1.01
Genu FA	0.3	0.91	1.00	0.99–1.01
MCP FA	0.2	0.91	1.00	0.99–1.01
SCP FA	2.6	0.46	1.01	1.00–1.02

Results for generalized estimating equations

*ATR* anterior thalamic radiation, *CST* cortico-spinal tract, *SCP* superior cerebellar peduncle, *MCP* midcerebellar peduncle, *q* false-discovery rate adjusted *p* value, *e* ^*B*^ exponentiated regression coefficient representing percent of increase in score for every hundredth unit increase in fractional anisotropy; *CI* 95% confidence interval, *RBS-R* Repetitive Behavior Scale-Revised, *SEQ* Sensory Experiences Questionnaire

### Exploratory analysis of brain and behavior interaction among high-risk infants

Although FDR corrected results for HR-Neg indicated no statistically significant results, we were nonetheless intrigued that the direction of effects for HR-Neg was the reverse of those observed in HR-ASD. We therefore conducted a limited set of exploratory analyses to more fully interrogate the effect size estimates observed for the genu and splenium of the corpus callosum in relation to total RBS-R scores.

First, we explored RBS-R results for the genu and splenium in HR-Neg by examining lower- and higher-order RRBs derived from the RBS-R. For the genu, we found that the relationship was strongest for lower-order (χ^2^ = 11.7, *p* = 0.001, *e* ^B^ = 0.90, 95% CI [0.84, 0.96]) versus higher-order behaviors (χ^2^ = 1.0, *p* = 0.34, *e* ^B^ = 0.97, 95% CI [0.92, 1.03]). Results were similar for the splenium: lower-order (χ^2^ = 8.3, *p* = 0.004, *e* ^B^ = 0.93, 95% CI [0.89, 0.98]) and higher order χ^2^ = 6.2, *p* = 0.01, *e* ^B^ = 0.95, 95% CI [0.91, 0.99].

**Fig. 2:**
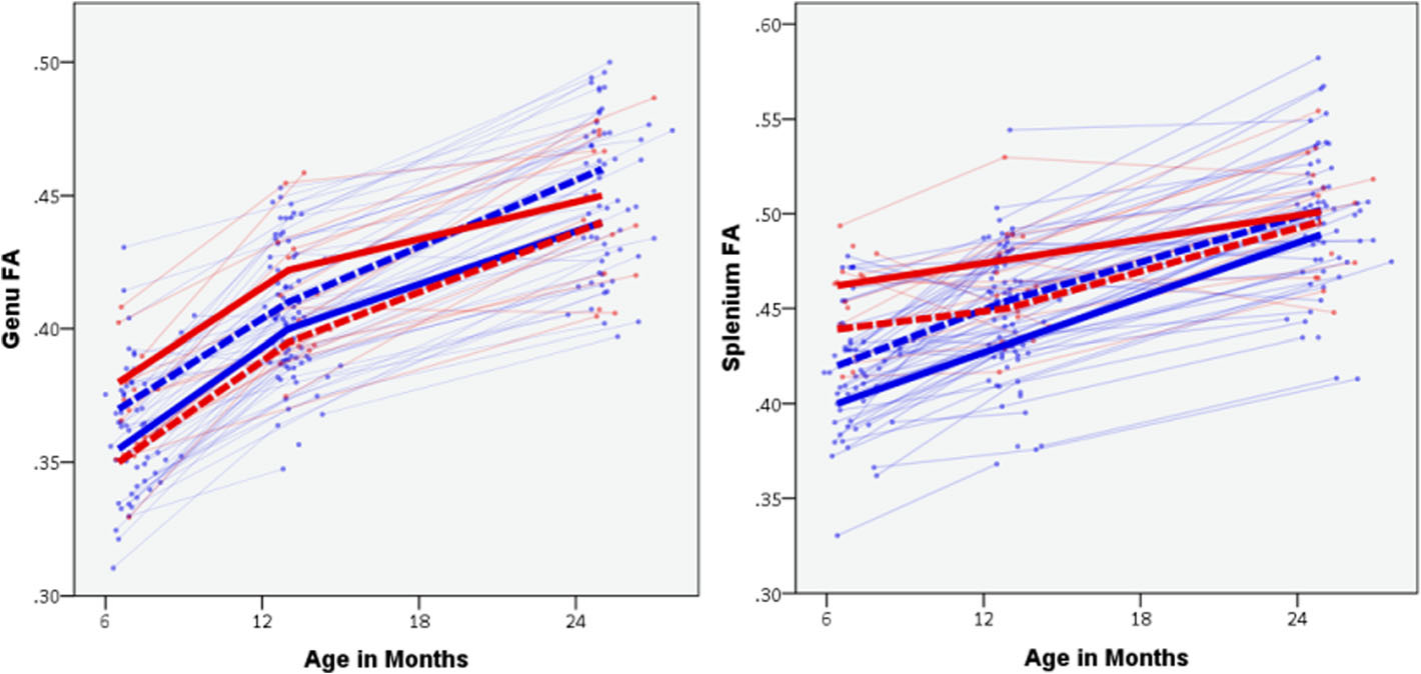
Trajectories of genu and splenium FA development by ASD diagnosis. *Red* signifies high-risk children diagnosed with ASD at age 2. *Blue* signifies high-risk children without ASD at age 2. Bolded lines represent *upper* and *lower* quartile groups based on total RBS-R score at age 2. *Solid bold* lines represent upper quartile of RBS-R scores. *Dashed* lines represent lower quartile of RBS-R scores. Quartile groups are presented solely to provide a visualization of the interaction effect between diagnostic status and FA on repetitive behavior measured at age 2, wherein higher FA was associated with more repetitive behavior for children with ASD, but was associated with less repetitive behavior for children without a diagnosis

As a direct test of the inverse relationship of genu/splenium FA and repetitive behavior scores observed between HR-ASD and HR-Neg groups, we examined the interaction of diagnostic group and genu/splenium FA on total RBS-R scores in generalized estimating equation models that included both HR-ASD and HR-Neg participants controlling for sex and age. The interaction of diagnostic group on FA was significant for the genu (χ^2^ = 4.3, *p* = 0.039) and splenium (χ^2^ = 4.8, *p* = 0.029). The interaction was also significant at the six-month time point in relation to later RBS-R score for the genu (χ^2^ = 10.0, *p* = 0.002) but not splenium (χ^2^ = 3.3, *p* = 0.07). Visualizations of these interactions are provided in Fig. 2. To determine whether interaction effects were specific to repetitive behavior in relation to corpus callosum FA, we expanded our analysis to include all targeted pathways as well as SEQ scores in a longitudinal model including both HR-ASD and HR-Neg. For RBS-R score, we observed an additional significant interaction for SCP FA (χ^2^ = 4.0, *p* = 0.046). For the SEQ scores, we observed a significant interaction between diagnostic status and genu FA (χ^2^ = 4.8, *p* = 0.029), with effects for the MCP and SCP just above the critical alpha value (*p* = 0.067 and 0.076). All other interactions were not statistically significant (see Additional file 1: Table A6). When diagnostic status was not accounted for in the above models, there were no significant predictive effects of brain on behavior.

Given the inverse relationship observed between brain and behavior by diagnostic outcome, as a final step we explored whether FA was predictive of diagnostic status. At age 6 months, FA in the ATR, CST, Genu, and MCP did not significantly predict diagnostic outcome (all *p* > 0.10). However, diagnostic outcome was significantly predicted by FA at age 6 months for the splenium (χ^2^ = 18.4, *p* < 0.001, *e* ^B^ = 1.44, 95% CI [1.22, 1.71; PPV = 65%, NPV = 84%]) and SCP (χ^2^ = 20.2, *p* < 0.001, *e* ^B^ = 1.81, 95% CI [1.40, 2.35]; PPV = 68%, NPV = 85%). Consistent with previous results [6], these findings suggest that higher FA at age 6 months is associated with increased risk for a later diagnosis of ASD.

## Discussion

We found that structural properties of cerebellar and corpus callosum white matter in infancy were associated with later-emerging restricted and repetitive behaviors (RRBs) and responsiveness to sensory stimuli in familial high-risk infants who developed ASD. Effects were uniformly characterized by a positive association between FA and symptom severity at age 2. The brain-behavior relationships we observed appeared specific to RRBs and sensory responsiveness given the absence of any association with social deficits. We also identified a strong positive association between RRBs and sensory response patterns measured at age 2 that remained even when controlling for cognitive ability or social symptom severity. Together these results suggest alignment between these classes of behavior in both outward manifestation and in underlying neurobiology early in the course of ASD.

To follow-up our primary results, we extended our analyses to high-risk infants who did not receive a diagnosis of ASD. As with HR-ASD, we observed that RRBs were significantly correlated with patterns of sensory responsiveness. We did not observe any statistically significant brain-behavior relationships after correction for multiple comparisons. However, effect size estimates suggested that genu and splenium FA may be associated with later RRBs in the HR-Neg group. Curiously, the direction of this effect was reversed, with FA *negatively* associated with repetitive behaviors at age 2 in HR-Neg. Given this unusual result, we conducted a limited set of exploratory analyses, the results of which suggest that diagnostic status may exert a moderating effect on the relationship between FA and RRB. This adds to previous reports of disordinal interactions in brain and behavior between infants who did and did not receive a diagnosis of ASD [4, 5]. For example, we have previously identified a disordinal interaction between infants who developed ASD and typically developing controls in the relationship of visual orienting latency to structural connectivity in the splenium [4]. Moreover, higher FA associated with a diagnosis of ASD early in life has been reported by several independent groups [5, 6, 60–63]. The preliminary data reported here further suggest that white matter development in infants with ASD may be fundamentally altered relative to unaffected children. As opposed to its connotation in DTI studies of typically developing children, higher FA may reflect pernicious neurodevelopmental effects unique to autism during infancy. It is plausible that higher FA in babies who develop ASD reflects underlying differences in axon structure related to axon refinement, caliber, or packing density. Further work is required to determine the timing of this phenomenon and whether a subgroup of children is driving it. It will also be necessary to explore alternative explanations, such as the possibility that effects observed through DTI are secondary to some unaccounted variable.

Our findings align with previous work identifying a role for the cerebellum in repetitive behavior [18, 27–29] and sensory processing [64, 65]. The absence of a strong association between RRBs with striatal pathways overall was counter to what we expected based upon previous work and supports the specificity of our findings. In our view there, are two plausible explanations. The first involves potential limitations. Our ability to measure more nuanced pathways within the basal ganglia was limited by imaging resolution and immaturity of the infant brain. DTI data is but one means of pursuing brain-behavior relationships, and the absence of signal using this modality does not preclude other approaches revealing clearer striatal involvement.

With regard to the second possibility, it is plausible that cerebellar circuitry supports nascent restricted and repetitive behaviors in early childhood, with striatal circuits coming online as such behaviors become entrenched through experience [19, 66, 67] or as later-developing behaviors emerge. Indeed, much of what is known about the role of the striatum comes from data involving older children [12–15], adults [16–18], or adult animal models [10]. It is less clear what role striatal circuitry plays during infant development or how such circuitry might interact with cerebellar circuits during this period to support emerging function [39], such as the transition from reflexive to instrumental behavior [67]. There is some evidence that the cerebellum and basal ganglia co-develop with motor skill acquisition during infancy [68] and that both share crucial roles in processing output from the cortex —mutually supportive roles which are substantially less segregated than once thought [22, 69].

Cerebellar and striatal circuits innervate similar frontal cortical regions [70, 71], which are in turn connected by the genu of the corpus callosum. The indirect involvement of the genu in both cerebellar and striatal circuits may in part explain our finding that genu FA strongly predicts RRBs and sensory responsiveness. However, we did find some evidence implicating the splenium as well, suggesting a possibly more general role for the corpus callosum in these emerging behaviors. Though less often the focus of such studies, there is precedence for corpus callosum involvement with RRBs associated with ASD [5] and other psychiatric conditions [72, 73]. There is also the strong possibility that our findings reflect biobehavioral mechanisms specific to early development. That is, the corpus callosum may play a unique functional role during infancy, complicating attempts to interpret its involvement through the lens of adult models. A direct test of this premise requires longitudinal follow-up into later childhood to characterize how the brain-behavior relationships observed herein change over time.

Owing to revised diagnostic criteria, we were interested in the co-occurrence of sensory responsiveness and RRBs among toddlers with ASD. We identified strong correlations between these behaviors even with social symptom severity or general developmental level held constant. This adds to a limited body of evidence supporting a relationship between RRBs and sensory responsiveness [30–32]. As conventionally defined, RRBs outwardly share in common two defining elements: they are relatively invariant in topography and occur repeatedly. The manner in which such behaviors are linked to atypical responses to sensory stimuli is a matter which requires further study. Do measures of these behaviors reflect different aspects of some primary phenomenon? Do repetitive behaviors and unusual responses to sensory stimuli *belong* together within a plausible, falsifiable theoretical framework [33]? Are existing concepts and terminology related to sensory responsiveness in ASD overly reductionist? While the present results suggest substantial overlap between RRBs and patterns sensory responsiveness in relation to one another and to underlying neurobiology, these critical questions ultimately remain unresolved.

There are several limitations which merit discussion. Our analyses relied on parent report measures, which provide an indirect assessment of behavior. Alternative measures may yield more accurate data and allow inclusion of a low-risk contrast group. Future work might also go beyond course divisions of behavioral domains, such as lower/higher-order RRBs, to examine fine-grained brain-behavior relationships. In this study, bilateral fiber pathways were averaged to yield a single estimate. While supplementary analyses indicated a general absence of lateralization, there were some marginal effects which may warrant closer investigation (Additional file 1: Tables A4-A5). The present analyses were hypothesis-driven and were focused on a specific set of white matter pathways. Follow-up work might elect to further vet regions such as the corpus callosum and cerebellum as well as consider alternate regions. Although diagnoses made at age 2 years show strong stability over time [1, 7], ASD is a heterogeneous disorder and we expect the present findings may be representative only of samples enrolled and assessed with similar procedures [74]. Following infants past early childhood might afford the opportunity to better characterize developmental phenotypic heterogeneity and chart dynamic brain-behavior relationships over time. Lastly, our exploratory analyses into possible interaction effects among all HR participants were limited to a specific set of white matter pathways and behavioral measures. A more comprehensive investigation into potential interactions between diagnostic status and FA development on RRBs and other behaviors core to ASD is needed.

## Conclusions

We observed that restricted and repetitive behaviors and unusual responses to sensory stimuli co-occur in toddlers with ASD and that these behaviors are associated with the structural properties of callosal and cerebellar white matter circuits measured during infancy and toddlerhood. These brain-behavior relationships were remarkably specific, suggesting a possible neurobiological mechanism wherein atypical neural development in infancy precedes the emergence of core autistic features within the domain of restricted and repetitive behaviors. Identifying pre-symptomatic markers of later behavior in ASD also affords the possibility of developing enhanced approaches to screening and preventative interventions.

## Additional files

Additional file 1: Table A1. Longitudinal model results for axial and radial diffusivities with repetitive behavior and sensory responsiveness, HR-ASD. Table A2 Axial and radial diffusivities at age 6 months predicting repetitive behavior and sensory responsiveness measured at age 2 years, HR-ASD. Table A3 Nonparametric correlations for subscales derived from the RBS-R and SEQ among HR-Neg. Table A4 Longitudinal model results for fractional anisotropy of bilateral pathways with repetitive behavior and sensory responsiveness, HR-ASD. Table A5 Fractional anisotropy of bilateral pathways at age 6 months predicting repetitive behavior and sensory responsiveness measured at age 2 years, HR-ASD. Table A6 Interaction of diagnostic status and fractional anisotropy on behavior. (DOCX 27 kb)
Additional file 2: Figure S1. Scatterplots of FA in select white matter pathways at age 6 months with RBS-R and SEQ scores. Scatterplots show relationship of FA in two pathways measured at age 6 months (genu, cortico-spinal tract) with total Repetitive Behavior Scale-Revised (RBS-R) and Sensory Experiences Questionnaire (SEQ) scores at age 24 months. Panels **a** and **c** were selected to provide visualization of a significant relationship between brain-and behavior in children with ASD (HR-ASD, in red). Panels **b** and **d** provide examples of a non-significant relationship (RBS-R and SEQ, respectively). High-risk children without ASD (HR-Neg) are shown in blue. Note that linear trend lines are for visualization purposes only and were not the basis of statistical modeling. (TIF 191 kb)

## Abbreviations

ADOS: Autism Diagnostic Observation Schedule
ASD: Autism spectrum disorder
ATR: Anterior thalamic radiation
CST: Cortical spinal tract
DT-MRI: Diffusion tensor magnetic resonance imaging
FA: Fractional anisotropy
GEE: Generalized estimating equation
GLM: Generalized linear model
HR: High risk
MCP: Midcerebellar peduncle
RBS-R: Repetitive Behavior Scale-Revised
RRBs: Restricted and repetitive behaviors
SCP: Superior cerebellar peduncle
SEQ: Sensory Experiences Questionnaire
