# Supplementary material for: Neural circuitry at age 6 months associated with later repetitive behavior and sensory responsiveness in autism
Source: Mol Autism. 2017 Mar 4;8:8. doi: 10.1186/s13229-017-0126-z (PMC5351210; doi:10.1186/s13229-017-0126-z)
Supplement: Additional file 1: Table A1. — Longitudinal model results for axial and radial diffusivities with repetitive behavior and sensory responsiveness, HR-ASD. Table A2 Axial and radial diffusivities at age 6 months predicting repetitive behavior and sensory responsiveness measured at age 2 years, HR-ASD. Table A3 Nonparametric correlations for subscales derived from the RBS-R and SEQ among HR-Neg. Table A4 Longitudinal model results for fractional anisotropy of bilateral pathways with repetitive behavior and sensory responsiveness, HR-ASD. Table A5 Fractional anisotropy of bilateral pathways at age 6 months predicting repetitive behavior and sensory responsiveness measured at age 2 years, HR-ASD. Table A6 Interaction of diagnostic status and fractional anisotropy on behavior. (DOCX 27 kb) [file 13229_2017_126_MOESM1_ESM.docx]

**Additional file 1**

**Contents: Tables A1 – A6**

**Table A1. Longitudinal model results for axial and radial diffusivities with repetitive behavior and sensory responsiveness, HR-ASD.**

|  | **Axial diffusivity** | |  | **Radial diffusivity** | |
| --- | --- | --- | --- | --- | --- |
|  | ***X*^2^** | **Ρ** |  | ***X*^2^** | **Ρ** |
| **RBS-R total** |  |  |  |  |  |
| ATR | 0.1 | 0.71 |  | 0.5 | 0.49 |
| CST | 0.0 | 0.84 |  | 0.0 | 0.93 |
| Genu | 9.0 | 0.003 |  | 5.4 | 0.02 |
| MCP | 1.5 | 0.22 |  | 2.9 | 0.09 |
| SCP | 0.5 | 0.49 |  | 4.8 | 0.03 |
| **SEQ total** |  |  |  |  |  |
| ATR | 0.9 | 0.35 |  | 0.2 | 0.67 |
| CST | 0.2 | 0.63 |  | 0.1 | 0.74 |
| Genu | 4.1 | 0.04 |  | 1.7 | 0.19 |
| MCP | 0.5 | 0.48 |  | 1.4 | 0.23 |
| SCP | 0.2 | 0.65 |  | 0.7 | 0.40 |

ATR = anterior thalamic radiation; CST = cortico-spinal tract; SCP = superior cerebellar peduncle; MCP = midcerebellar peduncle

RBS-R = Repetitive Behavior Scale, Revised; SEQ = Sensory Experiences Questionnaire

**Table A2. Axial and radial diffusivities at age 6 months predicting repetitive behavior and sensory responsiveness measured at age 2 years, HR-ASD.**

|  | **Axial diffusivity** | |  | **Radial diffusivity** | |
| --- | --- | --- | --- | --- | --- |
|  | ***X*^2^** | **Ρ** |  | ***X*^2^** | **Ρ** |
| **RBS-R total** |  |  |  |  |  |
| ATR | 0.1 | 0.83 |  | 0.4 | 0.55 |
| CST | 0.1 | 0.82 |  | 0.1 | 0.73 |
| Genu | 0.3 | 0.58 |  | 10.8 | 0.001 |
| MCP | 2.3 | 0.13 |  | 2.4 | 0.12 |
| SCP | 0.1 | 0.79 |  | 1.7 | 0.20 |
| **SEQ total** |  |  |  |  |  |
| ATR | 0.0 | 0.92 |  | 3.2 | 0.08 |
| CST | 0.0 | 0.84 |  | 0.7 | 0.40 |
| Genu | 1.4 | 0.23 |  | 5.1 | 0.02 |
| MCP | 1.0 | .033 |  | 1.7 | 0.19 |
| SCP | 2.5 | 0.12 |  | 0.1 | 0.76 |

ATR = anterior thalamic radiation; CST = cortico-spinal tract; SCP = superior cerebellar peduncle; MCP = midcerebellar peduncle

RBS-R = Repetitive Behavior Scale, Revised; SEQ = Sensory Experiences Questionnaire

**Table A3. Nonparametric correlations for subscales derived from the RBS-R and SEQ among HR-Neg.**

|  | **SEQ Total** | **SEQ Hypo** | **SEQ Hyper** | **SEQ Sensory Seeking** |
| --- | --- | --- | --- | --- |
| **RBS-R lower-order** |  |  |  |  |
| *r*_s_ | 0.47*** | 0.47*** | 0.38*** | 0.36*** |
| 95% CI | 0.34, 0.59 | 0.33, 0.60 | 0.24, 0.53 | 0.20, 0.50 |
| **RBS-R higher-order** |  |  |  |  |
| *r*_s_ | 0.42*** | 0.38*** | 0.38*** | 0.27** |
| 95% CI | 0.27, 0.54 | 0.23, 0.51 | 0.24, 0.52 | 0.11, 0.42 |

RBS-R = Repetitive Behavior Scale, Revised; SEQ = Sensory Experiences Questionnaire

*** *p* < 0.001, ** *p* < 0.01, * *p* < .05

**Table A4. Longitudinal model results for fractional anisotropy of bilateral pathways with repetitive behavior and sensory responsiveness, HR-ASD.**

|  | **RBS-R total** | |  | **SEQ total** | |
| --- | --- | --- | --- | --- | --- |
|  | ***X*^2^** | **Ρ** |  | ***X*^2^** | **Ρ** |
| **ATR L** | 1.4 | 0.23 |  | 0.0 | 0.82 |
| **ATR R** | 2.7 | 0.10 |  | 1.3 | 0.26 |
| **CST L** | 0.0 | 0.97 |  | 0.0 | 0.89 |
| **CST R** | 0.4 | 0.51 |  | 0.0 | 0.97 |
| **SCP L** | 5.8 | 0.016 |  | 5.4 | 0.02 |
| **SCP R** | 4.2 | 0.04 |  | 3.2 | 0.07 |

ATR = anterior thalamic radiation; CST = cortico-spinal tract; SCP = superior cerebellar peduncle

RBS-R = Repetitive Behavior Scale, Revised; SEQ = Sensory Experiences Questionnaire

**Table A5. Fractional anisotropy of bilateral pathways at age 6 months predicting repetitive behavior and sensory responsiveness measured at age 2 years, HR-ASD.**

|  | **χ^2^** | **Ρ** |
| --- | --- | --- |
| **RBS-R total** |  |  |
| ATR L | 0.5 | 0.48 |
| ATR R | 0.1 | 0.75 |
| CST L | 0.3 | 0.56 |
| CST R | 0.1 | 0.81 |
| SCP L | 0.4 | 0.54 |
| SCP R | 0.6 | 0.42 |
| **SEQ total** |  |  |
| ATR L | 1.5 | 0.22 |
| ATR R | 3.0 | 0.08 |
| CST L | 3.9 | 0.05 |
| CST R | 0.2 | 0.64 |
| SCP L | 3.7 | 0.06 |
| SCP R | 3.4 | 0.07 |

ATR = anterior thalamic radiation; CST = cortico-spinal tract; SCP = superior cerebellar peduncle

RBS-R = Repetitive Behavior Scale, Revised; SEQ = Sensory Experiences Questionnaire

**Table A6**. Interaction of diagnostic status and fractional anisotropy on behavior.

| **Variable** | **χ^2^** | **Ρ** |
| --- | --- | --- |
| **Total RBS-R** |  |  |
| ATR FA | 0.6 | 0.45 |
| CST FA | 0.1 | 0.75 |
| Genu FA | 4.3 | 0.04 |
| MCP FA | 2.8 | 0.10 |
| SCP FA | 4.0 | 0.05 |
| Splenium FA | 4.8 | 0.03 |
| **Total SEQ** |  |  |
| ATR FA | 0.1 | 0.74 |
| CST FA | 0.0 | 0.99 |
| Genu FA | 4.8 | 0.03 |
| MCP FA | 3.4 | 0.07 |
| SCP FA | 3.1 | 0.08 |
| Splenium | 0.1 | 0.71 |

Results for generalized estimating equations

ATR = anterior thalamic radiation; CST = cortico-spinal tract; SCP = superior cerebellar peduncle; MCP = midcerebellar peduncle; RBS-R = Repetitive Behavior Scale, Revised; SEQ = Sensory Experiences Questionnaire
