# Supplementary figures and images for: Neural circuitry at age 6 months associated with later repetitive behavior and sensory responsiveness in autism
Source: Mol Autism. 2017 Mar 4;8:8. doi: 10.1186/s13229-017-0126-z (PMC5351210; doi:10.1186/s13229-017-0126-z)

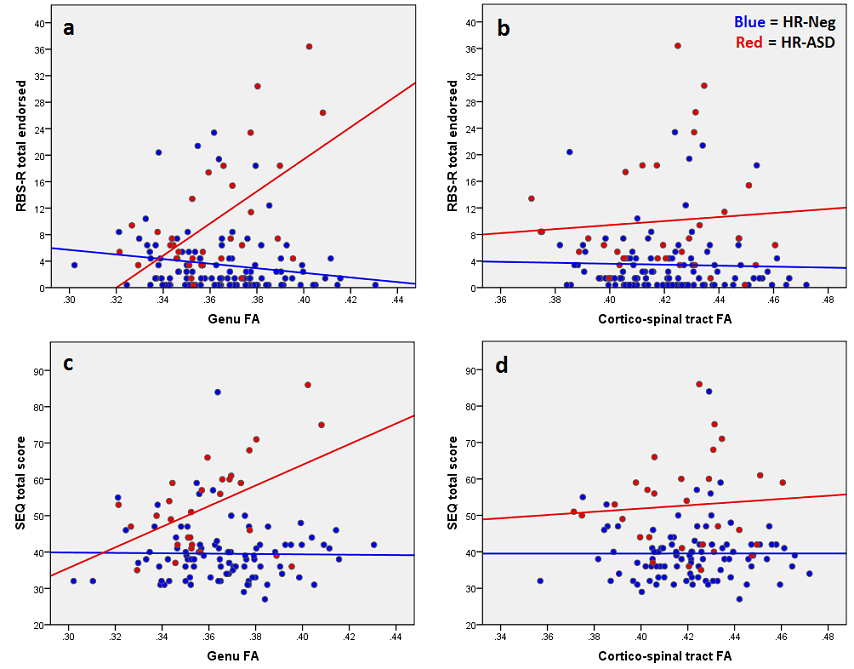

Supplement: Additional file 2: Figure S1. — Scatterplots of FA in select white matter pathways at age 6 months with RBS-R and SEQ scores. Scatterplots show relationship of FA in two pathways measured at age 6 months (genu, cortico-spinal tract) with total Repetitive Behavior Scale-Revised (RBS-R) and Sensory Experiences Questionnaire (SEQ) scores at age 24 months. Panels a and c were selected to provide visualization of a significant relationship between brain-and behavior in children with ASD (HR-ASD, in red). Panels b and d provide examples of a non-significant relationship (RBS-R and SEQ, respectively). High-risk children without ASD (HR-Neg) are shown in blue. Note that linear trend lines are for visualization purposes only and were not the basis of statistical modeling. (TIF 191 kb) [file 13229_2017_126_MOESM2_ESM.tif]
